# Vertical split-ring resonator based anomalous beam steering with high extinction ratio

**DOI:** 10.1038/srep11226

**Published:** 2015-06-08

**Authors:** Wei-Lun Hsu, Pin Chieh Wu, Jia-Wern Chen, Ting-Yu Chen, Bo Han Cheng, Wei Ting Chen, Yao-Wei Huang, Chun Yen Liao, Greg Sun, Din Ping Tsai

**Affiliations:** 1 Department of Physics, National Taiwan University, Taipei 10617, Taiwan; 2 Research Center for Applied Sciences, Academia Sinica, Taipei 11529, Taiwan; 3 Department of Engineering, University of Massachusetts Boston, Boston, Massachusetts 02125, U.S.A

## Abstract

Metasurfaces created artificially with metal nanostructures that are patterned on surfaces of different media have shown to possess “unusual” abilities to manipulate light. Limited by nanofabrication difficulties, so far most reported works have been based on 2D metal structures. We have recently developed an advanced e-beam process that allowed for the deposition of 3D nanostructures, namely vertical split-ring resonators (VSRRs), which opens up another degree of freedom in the metasurface design. Here we explore the functionality of beam steering with phase modulation by tuning only the vertical dimension of the VSRRs and show that anomalous steering reflection of a wide range of angles can be accomplished with high extinction ratio using the finite-difference-time-domain simulation. We also demonstrate that metasurfaces made of 3D VSRRs can be made with roughly half of the footprint compared to that of 2D nano-rods, enabling high density integration of metal nanostructures.

Metamaterials, the artificial structures with plasmonic sub-wavelength scale structures, promising novel and exotic electromagnetic phenomena not found in nature, such as negative refraction^1^,^2^,^3^, super-resolution^4^,^5^,^6^ and cloaking^7^,^8^, have attracted much attention. Nearly all these “unusual” phenomena of metamaterials are connected to the localized surface plasmon resonances (LSPR)^9^ associated with the subwavelength metal structures. Unfortunately, those metamaterials calling for construction of multi-layer nanostructures have been very challenging to fabricate and even when they are made the extraordinary properties that require light penetration into the medium are difficult to observe because of the tremendous optical loss associated with the metal typically used as the constituent nanostructures. As a way to alleviate these insurmountable barriers, recently a subcategory of metamaterials known as metasurfaces has emerged where only a single layer of metal nanostructures deposited on the surface of a dielectric medium is utilized to realize those “unusual” abilities to manipulate light. For instance, the extraordinary Snell’s law was demonstrated using the extra momentum contributed by a metasurface^10^. These phenomena have been successfully employed in a range of practical applications including light steering^11^,^12^,^13^, flat lenses^14^,^15^, polarization converter^16^ and hologram imaging devices^17^,^18^. The concept of reflective metasurfaces was recently proposed to improve the operation efficiencies of these devices^11^,^18^.

We have recently developed a high precision alignment technique that enables us to fabricate metasurfaces made of the vertical split-ring resonators (VSRRs)^19^,^20^,^21^ capable of both phase and reflection modulation by controlling the VSRR dimensions. In comparison with 2D SRRs where the tunings of LSPRs are achieved with variation of dimensions in the x-y plane of the metasurface, the VSRRS allows for phase and reflectance modulation by changing the heights of their prongs along z-direction, effectively providing an additional degree of freedom in design. Here we propose to use VSRRs as the basic building blocks to construct metasurface that reflects a normal incident light within telecommunication band to a direction tunable by design in violation of the conventional Snell’s law. The metasurface is patterned with periodical unit cells where each unit consisting of six Au VSRRs with gradient prong lengths sitting on fixed base. The unit cell period determines the reflection angle of light upon its incidence on the metasurface. This investigation is carried out with numerical simulation where we have used the periodical boundary conditions. Results indicate that a highly directional reflection can be achieved with the full-width-at-half-maximum (FWHM) angle of 2.9^o^ at *λ* = 1548 nm and the anomalous reflection signal shows the extinction ration as high as 31 relative to that of normal reflection. In comparison with the metasurface made of 2D metal nano-rods where the LSPR is modulated with rod length, our 3D-VSRR design with tuning of prong height has the advantage of covering the surface area with higher density of metal structures which is desirable for minimizing metasurface device size for applications in integrated photonics.

## Results and discussions

**Figure 1:**
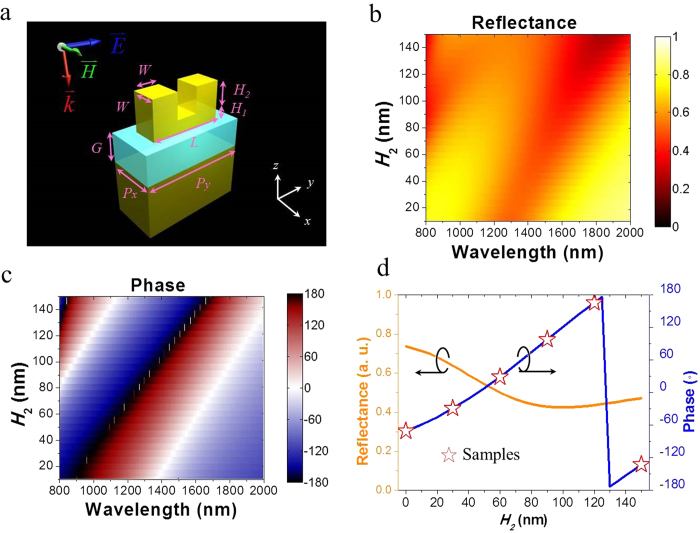
Reflectance and phase shift in isolated VSRR structures. (**a**) Schematic diagram for VSRR with structural parameters: *L* = 170 nm, *W* = 60 nm, *H*_1_ = 30 nm, *P*_*x*_ = 120  m, *P*_*y*_ = 250 nm, *G* = 70 nm, and prong height *H*_2_. Simulation of (**b**) reflectance and (**c**) phase shift with various *H*_*2*_ under y-polarized normal illumination of different wavelength. (**d**) Reflectance and phase shift as a function of *H*_*2*_ at λ = 1548 nm. Red stars indicate the chosen values of VSRR prong height *H*_2_ to be implemented in a unit cell.

The basic building block as shown in Fig. 1(a) is an Au VSRR which is composed of a base rod and two prongs standing on its two ends. The VSRRs are deposited on a SiO_2_ layer (*G* = 70 nm) over an Au mirror. The thin SiO_2_ spacer is necessary for the coupling between VSRRs and the bottom Au mirror to achieve strong excitation^22^ of the LSPR and broader phase modulation^23^. Each VSRR has its base rod fixed with dimensions of *L* = 170 nm, *W* = 60 nm, and *H*_1_ = 30 nm, while the height (*H*_2_) of the prongs is varied to obtain the desired phase modulation. Each VSRR occupies an area of 120 × 250 nm^2^. The finite-difference time domain (FDTD) based commercial software CST is used to simulate the reflectance and phase shift by a 2D array of such VSRRs. The VSRRs are excited with a light source polarized along the VSRR base length (y-direction marked in Fig. 1(a)). The reflectance and phase shift vs. VSRR prong height (*H*_2_) and excitation wavelength (λ) are shown in Fig. 1(b,c), respectively, where we can see that reflectance variation is far more modest than that of the phase shift, a desirable scenario for the beam steering application for preserving its intensity. This is better viewed in Fig. 1(d) at a single wavelength of λ = 1548 nm, for the range of prong height *H*_2_ that yields phase modulation of 2π the reflectance only varies within 0.45 to 0.75. The phase modulation curve calculated in Fig. 1(d) needs to be digitized when implemented on metasurface for beam steering. For wave front reconstruction under the assumption of uniform reflectance, 2π phase modulation with a constant phase gradient is important. We have chosen to use six equally spaced phase modulation points separated by 60^°^ corresponding to prong heights of *H*_2_ = 30, 60, 90, 120, 150, and 0 nm as shown in Fig. 1(d). We could have designed finer phase modulation steps with smaller VSRR height changes, but this would present a significant challenge in future fabrication of such metasurface.

**Figure 2:**
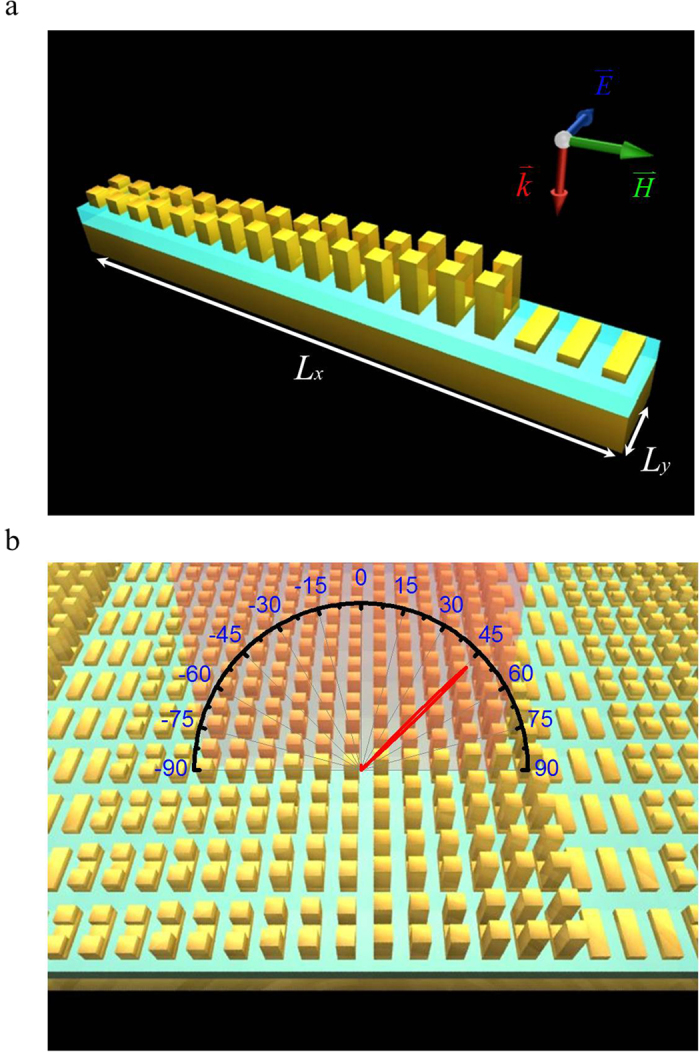
Schematic of VSRR-based metasurface. (**a**) Schematic of a unit cell consisting of 18 VSRR of equal base but six different prong heights: 30, 60, 90, 120, 150, and 0 nm, with three VSRRs of equal dimensions. Each unit cell occupies *L*_*x*_ = 2160 nm and *L*_*y*_ = 250 nm. (**b**) Illustration of the VSRR-based metasurface beam steering.

We subsequently use these VSRRs of six different heights to construct a unit cell with the necessary period to steer a normal incident beam of a particular wavelength onto a pre-determined angle. Figure 2(a) shows the schematic of a VSRR based unit cell occupying an area of L_*x*_ × L_*y*_ = 2160 × 250 nm^2^. Such a unit cell is repeated along x- and y-directions to form the functional metasurface where the long period of 2160 nm is chosen to yield a steering angle of 45^°^ for the normal incident light of λ = 1548 nm as shown in Fig. 2(b). It takes 18 VSRRs to fill up a unit cell (Fig. 2(a)) in which six sets of three VSRRs of same height are arranged to obtain the phase modulations in Fig. 1(d). In order for a normal incident beam to be redirected to 45^°^ (Fig. 2(b)) according to the generalized Snell’s law, the amount of in-plane wave-vector that needs to be provided by the metasurface is 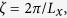
where 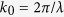 is the wavevector in free space. It follows then that such a metasurface will also steer light with an arbitrary incident angle *θ*_*i*_ to the reflection angle *θ*_*r*_ according to 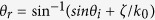.

**Figure 3:**
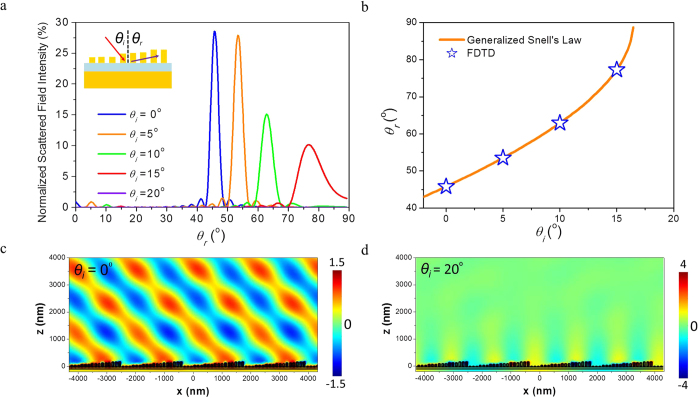
Scattered field of VSRR-based matasurface. (**a**) Angular dependence of the reflected field intensity under various incident angles at λ = 1548 nm. (**b**) FDTD results (blue stars) and generalized Snell’s Law (orange line). Simulation of the wave front (y-component of the electric field) plotted in the x-z plane for incident angle **(c**) θ_i_ = 0° and (**d**) 20°.

Figure 3(a) shows the normalized field intensity of reflection at 1548 nm under various incident angle (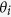 = 0°, 5°, 10°, 15°, and 20°). The reflection angle 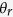 increases from 45° to 77° as the incident angle 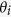 varied from 0° to 15°, consistent with our design parameters. Small amount of scattered light can be seen within the region of 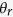 < 40° as a result of unmodulated. FWHM angle of 2.9° around the reflection angle of 45° is obtained for the normal incidence and the intensity of reflection at 45^°^ is 31 times stronger than that of normal reflection, suggesting that the VSRR-based metasurface is capable of steering light with a high extinction ratio. Figure 3(b) shows that the FDTD simulation result is good agreement with generalized Snell’s Law 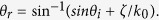 The simulation result of the reflected wave front propagating along 45° is displayed in Fig. 3(c) for the normal incident. As the incident angle is increased to 20°, no reflection can be observed as shown in Fig 3(a) as the incident light is being diffracted into a surface wave with its wave front normal to the metasurface as shown in Fig. 3(d).

**Figure 4:**
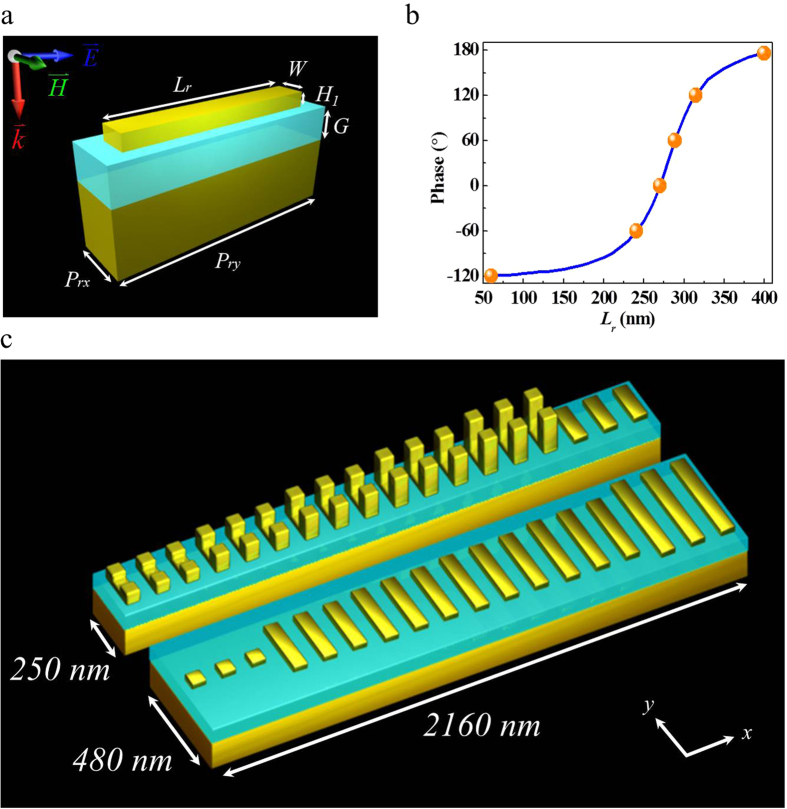
Footprint comparison. (**a**) Schematic of an Au nano-rod as basic building block for metasurface with dimensions of *W* = 60 nm, *H*_1_ = 30 nm, and length *L*_*r*_, sitting on a SiO_2_ layer of *G* = 70 nm over an Au mirror. Each nano-rod occupies an area of *P*_*rx*_* × P*_*ry*_ = 120 × 480 nm^2^. (**b**) Phase shift as function of rod length *L*_*r*_ at wavelength of 1548 nm. Orange points indicate the chosen lengths for the nano-rods to be used in a metasurface unit cell for beam steering. **(c**) Illustration of the VSRR-based (top) and nano-rod-based (bottom) metasurfaces for comparison of their footprint.

In comparison with metasurfaces made of the simplest metal nanostructures such as Au nano-rods, the VSRRs require a smaller footprint to perform the same functionalities. To illustrate this, we have simulated the optical response of 2D arrays made of Au nano-rods patterned on the same SiO_2_ layer of 70 nm thick on top of an Au mirror at λ = 1548 nm. As shown in Fig. 4(a), each Au nano-rod has a fixed width of *W* = 60 nm and thickness of *H*_1_ = 30 nm, and occupies an area of *P*_*rx*_ × *P*_*ry*_ = 120 × 480 nm^2^. Figure 4(b) shows the range of nano-rod length *L*_r_ required to obtain 2π phase shift. Similar to our VSRR design, we can once again choose to use six equally spaced phase modulation points (orange dots in Fig. 4(b)) separated by 60^°^ corresponding to nano-rod lengths of *L*_*r*_ = 60, 240, 270, 288, 314, and 400 nm for beam steering at 1548 nm. The same long period of 2160 nm is required for the unit cell in order to attain the same steering performance. Such a unit cell consisting of 18 nano-rods (six sets of three nano-rods of same length) is shown side by side with the VSRR unit cell in Fig. 4(c). It can be seen that while both structures have the same long period of 2160 nm for equal steering, the nano-rod unit cell requires a much greater short period (480 nm) along y-direction in order to accommodate the longer nano-rod lengths than that of the VSRR base (250 nm). It can be established that the footprint of the VSRR unit cell is roughly half of that of nano-rods, allowing for implementation of high density configuration for a range of metasurface-based applications.

## Conclusions

In summary, we have conducted FDTD simulation of reflectance and phase shift by a VSRR metasurface for beam steering. Fixing the base dimensions of VSRRs, we vary their prong heights to obtain 2π phase modulation. We subsequently use six-level phase modulation design to construct VSRR unit cells. The simulation results show that VSRR-based metasurface enables directional and high extinction ratio beam steering for normal incidence at the telecommunication wavelength *λ* = 1548 nm. In addition, the metasurface also diffracts incident light into a surface wave when its angle of incidence approaches a critical angle. In comparison with metasurface made of nano-rods for the same beam steering functionality, the VSRR unit cell can be made with roughly half of the footprint, enabling high density integration of metal nanostructures.

## Additional Information

**How to cite this article**: Hsu, W.-L. *et al.* Vertical split-ring resonator based anomalous beam steering with high extinction ratio. *Sci. Rep.* **5**, 11226; doi: 10.1038/srep11226 (2015).
